# Application of Bayesian Approach to Cost-Effectiveness Analysis of Antiviral Treatments in Chronic Hepatitis B

**DOI:** 10.1371/journal.pone.0161936

**Published:** 2016-08-30

**Authors:** Hua Zhang, Mingdong Huo, Jianqian Chao, Pei Liu

**Affiliations:** 1 Department of Medical Insurance, School of Public Health, Southeast University, Nanjing, Jiangsu, China; 2 Zhongda Hospital Affiliated to Southeast University, Nanjing, Jiangsu, China; 3 Department of Epidemiology and Biostatistics, School of Public Health, Southeast University, Nanjing, Jiangsu, China; 4 Key Laboratory of Environmental Medicine Engineering, Ministry of Education, School of Public Health, Southeast University, Nanjing, China; Centre de Recherche en Cancerologie de Lyon, FRANCE

## Abstract

**Background:**

Hepatitis B virus (HBV) infection is a major problem for public health; timely antiviral treatment can significantly prevent the progression of liver damage from HBV by slowing down or stopping the virus from reproducing. In the study we applied Bayesian approach to cost-effectiveness analysis, using Markov Chain Monte Carlo (MCMC) simulation methods for the relevant evidence input into the model to evaluate cost-effectiveness of entecavir (ETV) and lamivudine (LVD) therapy for chronic hepatitis B (CHB) in Jiangsu, China, thus providing information to the public health system in the CHB therapy.

**Methods:**

Eight-stage Markov model was developed, a hypothetical cohort of 35-year-old HBeAg-positive patients with CHB was entered into the model. Treatment regimens were LVD100mg daily and ETV 0.5 mg daily. The transition parameters were derived either from systematic reviews of the literature or from previous economic studies. The outcome measures were life-years, quality-adjusted lifeyears (QALYs), and expected costs associated with the treatments and disease progression. For the Bayesian models all the analysis was implemented by using WinBUGS version 1.4.

**Results:**

Expected cost, life expectancy, QALYs decreased with age. Cost-effectiveness increased with age. Expected cost of ETV was less than LVD, while life expectancy and QALYs were higher than that of LVD, ETV strategy was more cost-effective. Costs and benefits of the Monte Carlo simulation were very close to the results of exact form among the group, but standard deviation of each group indicated there was a big difference between individual patients.

**Conclusions:**

Compared with lamivudine, entecavir is the more cost-effective option. CHB patients should accept antiviral treatment as soon as possible as the lower age the more cost-effective. Monte Carlo simulation obtained costs and effectiveness distribution, indicate our Markov model is of good robustness.

## Introduction

Chronic hepatitis B (CHB) infection affecting 350 to 400 million people is a public health problem globally. About 112 million people in China are chronically infected with hepatitis B virus (HBV) [1,2]. CHB infection has severe long-term outcomes and could contribute to hepatocellular carcinoma (HCC) cases [3]. It is estimated that CHB is among the ten leading causes of death worldwide [4]. The cost of health care, resulting in loss of life and productivity therefore has remarkable impact on the society.

To completely eradicate HBV is the ultimate goal of CHB treatment, but antiviral treatments for CHB do not provide a complete cure except for rare cases [5]. Timely treatment can significantly prevent the progression of liver damage from HBV by slowing down or stopping the virus from reproducing. Lamivudine (LVD) was the first oral agent to be approved for the treatment of HBV infection in China [6]. According to a study, 19% of patients received treatment mainly because of misunderstanding or economic restrictions [7]. In 2005, entecavir (ETV) was approved by the US Food and Drug Administration as a new guanosine nucleoside analogue for HBV treatment. ETV was recommended by the guidelines as a first-line therapy for CHB patients in China [8]. However, the long-term therapy costs have not been taken into consideration. ETV has found clinical benefits over LVD in clinical studies [9–13]. According to clinical results, including the significant difference in HBV DNA reduction reported, ETV is more cost effective than LVD [14,15]. Markov model was used to evaluate the cost-effectiveness of ETV and LVD for long-term estimates in some studies [3,16,17]. Up to date, classical statistical approaches have been used in most Markov models analysis exclusively.

In this study, from a Bayesian perspective we implemented the cost-effectiveness analysis, Markov Chain Monte Carlo simulation methods was used for the relevant evidence input into the model to evaluate cost-effectiveness of ETV and LVD in Jiangsu, China, thus providing information to the public health system in the CHB therapy.

## Materials and Methods

### Model description

**Fig 1:**
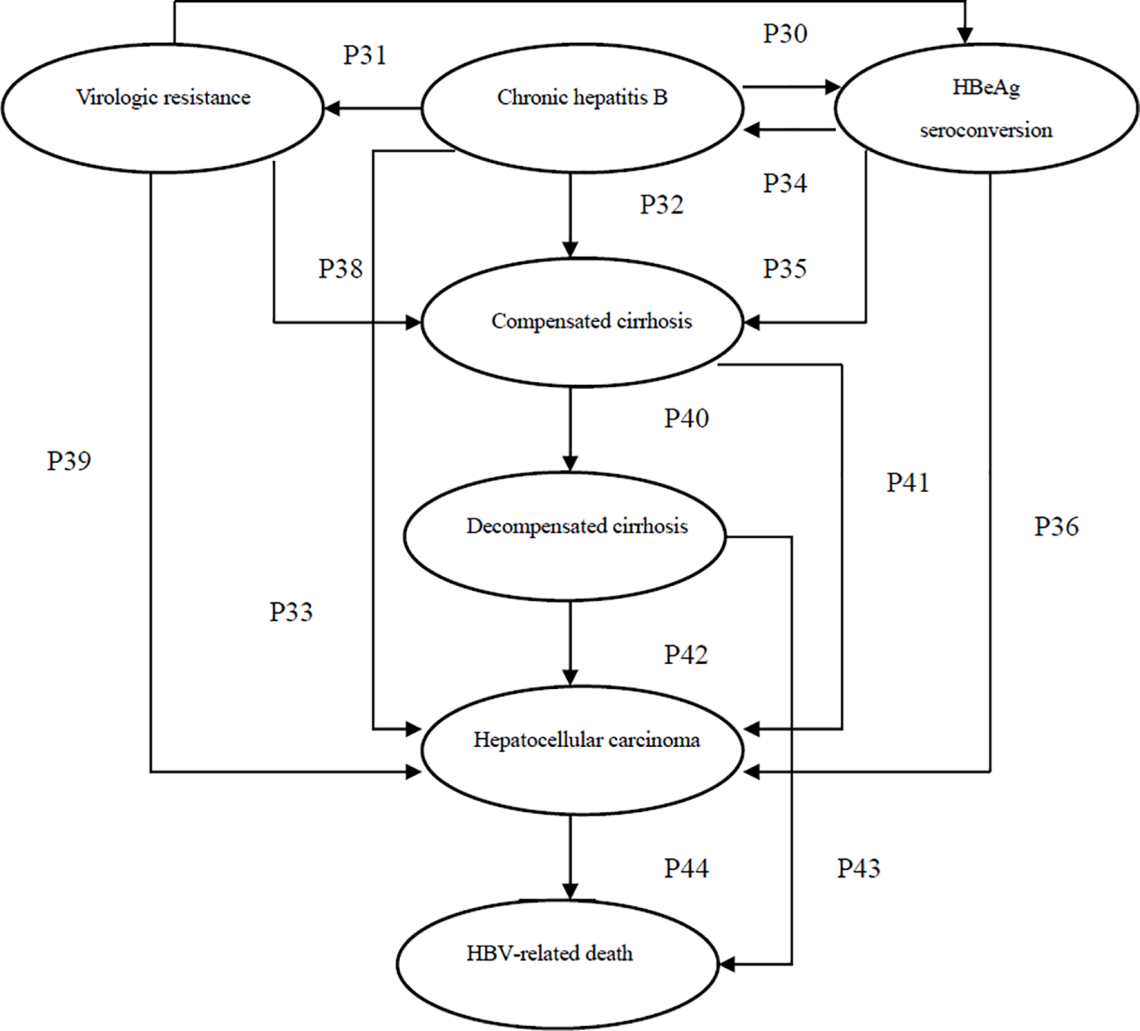
Markov diagram of health states and possible transitions between them.

To study the cost-effectiveness of using ETV and LVD treatment for CHB, an eight-stage Markov model was developed based on prior study [18]. The model of HBeAg-positive CHB was composed of eight mutually exclusive health states: chronic hepatitis B(CHB), HBeAg seroconversion, virologic resistance, compensated cirrhosis(CC), decompensated cirrhosis(DC), hepatocellular carcinoma(HCC), HBV-related death and general population death. It was assumed that in a Markov model individuals are always in one of a finite number of health states named Markov states and based on a series of transition probabilities health changes from state to state [19]. The transition probabilities depend only on the current health state that the individual is in and not on their previous states (the Markov assumption) [20]. The patient cohort enters the model in the CHB state. During each 1-year cycle, CHB patients either stayed in their assigned health state or changed to a new state. The Markov diagram of health states and possible transitions between them (see Fig 1). All states could lead to death, but general death was not shown in Fig 1.

The following were the main assumptions of our model

- Guidelines on the therapy of CHB recommend that treatment may be long term and could stop after HBeAg seroconversion and an additional 6 to 12 months of consolidation therapy to maximize the durability of treatment response [7,21]. The model employed the recommended treatment strategy,
- Differences of transition probabilities between LVD and ETV treatment include HBeAg seroconversion, virologic resistance and decompensated cirrhosis, other state transition probability between the two groups were assumed to be the same [22–27], since no significant difference was found in the literature,
- Only DC and HCC individuals could enter the HBV-related death state, because of relatively poor reported in the literature [28,29],
- After patients progressed to more severe disease states (CC, DC and HCC), transition probabilities and costs were associated with routine clinical practice.

A hypothetical cohort of 35-year-old HBeAg-positive patients with CHB was entered into the model. The model employed 42 yearly cycles on the basis of the Jiangsu life table. Treatment regimens included in the model were LVD 100mg daily, and ETV 0.5mg daily, all administered orally. In order to avoid overcomplicating the model, we excluded the possibility of dose reductions and treatment delays due to toxicities in the study. The outcome measures used in the model were life expectancy(years), quality-adjusted lifeyears (QALYs), and costs associated with the treatments and disease progression. Both cost and health outcomes in the model were discounted at 5% annually to allow for current values.

### Model parameters

Transitions between states are defined over a time frame (cycle length) of one year. The vector of state probabilities in cycle t = 1 is π1 = (1,0,0,0,0,0,0,0,0), The transition probability matrix for t = 2,…, 42 is given below (Table 1).Transition probabilities and proportions (Table 2) for Chinese CHB patients were derived from previous literature studies.

**Table 1:** Transition matrix of each state of chronic hepatitis B.

State	CHB	HBeAg	VR	CC	DC	HCC	HBVD	GD
CHB	1-P30-P31-P32-P33-λt	P30	P31	P32	0	P33	0	λt
HBeAg	P34	1-P34-P35-P36-λt	0	P35	0	P36	0	λt
VR	0	P37	1-P37-P38-P39-λt	P38	0	P39	0	λt
CC	0	0	0	1-P40-P41-λt	P40	P41	0	λt
DC	0	0	0	0	1-P42-P43-λt	P42	P43	λt
HCC	0	0		0	0	1-P44-λt	P44	λt
HBVD	0	0		0	0	0	0	1
GD	0	0		0	0	0	0	1

CHB = chronic hepatitis B, HBeAg = HBeAg seroconversion, VR = virologicresistence, CC = compensated cirrhosis, DC = decompensated cirrhosis, HCC = hepatocellular carcinoma, HBVD = HBV-related death, GD = general death.

**Table 2:** Probability of reaching each state of chronic hepatitis B.

Initial state	State reached	Base case	Reference	LVD	ETV	Reference
	HBeAg P30	0.077	[22]	0.18	0.19	[23]
CHB	VR P31			0.1188	0.03	[24,25]
	CC P32	0.044	[22]	0.02	0.007	[23]
	HCC P33	0.008	[26]	0.008	0.008	[26]
	Death	GD		GD	GD	
HBeAg	CHB P34	0.03	[22]	0.03	0.03	[22]
	CC P35	0.01	[22]	0.01	0.01	[22]
	HCC P36	0.003	[26]	0.003	0.003	[26]
VR	HBeAg P37	0.077	[26]	0.077	0.077	[26]
	CC P38	0.04	[26]	0.04	0.04	[26]
	HCC P39	0.0053	[26]	0.0053	0.0053	[26]
CC	DC P40	0.07	[27]	0.07	0.07	[27]
	HCC P41	0.034	[27]	0.034	0.034	[27]
DC	HCC P42	0.034	[27]	0.034	0.034	[27]
	HBVD P43	0.144	[28,29]	0.144	0.144	[28,29]
HCC	HBVD P44	0.40	[28,29]	0.40	0.40	[28,29]

CHB = chronic hepatitis B, HBeAg = HBeAg seroconversion, VR = virologicresistence, CC = compensated cirrhosis, DC = decompensated cirrhosis, HCC = hepatocellular carcinoma, HBVD = HBV-related death,GD = general death.

All drug costs were based on current official prices approved by the Jiangsu Municipal Bureau of Pricing. Annual cost of LVD (GlaxoSmithKline Ltd.) was US $865.39,ETV(Shanghai Squibb Company) was US $2006.57.Annual direct medical costs for managing these CHB-related disease states were derived from our previous study[30]. CHB: $4257.84, CC: $3910.69, DC: $6434.70, HCC: $4874.21. The costs were then converted to the December 31, 2012 US dollars (US $1 = CNY 6.2365).

The following Table 3 was the base health utilities of hepatitis B related disease in our study. The health state utilities for CHB patients were obtained primarily from previous studies in Jiangsu [23,31]. The utility for patients who achieved HBeAg seroconversion was assumed to be equivalent to that of uninfected respondents[26].

**Table 3:** Base-case health utilities.

Disease state	Quality of life	References
Chronic hepatitis B	0.795	[31]
HBeAg seroconversion	0.99	[26]
Virologicresistence	0.795	[31]
Compensated cirrhosis	0.695	[31]
Decompensated cirrhosis	0.661	[31]
Hepatocellular carcinoma	0.672	[31]
Death	0	[31]

### Analysis

WinBUGS1.4version was used for the Bayesian analysis. Monte Carlo simulation was adopted to sample from the specified ranges or distributions, whereas for the Bayesian model Markov Chain Monte Carlo(MCMC) simulation was implemented. For the Bayesian analyses, following preliminary test runs, an initial run of 10000 iterations was carried out as a ‘burn-in’ to reach convergence and inferences were based on 10000 iterations. (WinBUGS entire code is shown in S1 Code).

## Results

### Cost-effectiveness of alternative strategies

Table 4 showed expected costs, life expectancy, quality-adjusted lifeyears decreased with age. Cost-QALYs increased with the age group. Expected cost of ETV was less than LVD, while life expectancy and quality-adjusted life years were higher than that of LVD, ETV was more cost-effective option.

**Table 4:** Results of alternative strategies: costs, life expectancy, quality-adjusted life years (QALYs) gained.

Strategy	Subgroup	Cost($,C)	Life Expectancy (years, EL)	QALYs	C/EL	C/QALYs
LVD	35 yrs -	55690.08	15.45	13.54	3604.54	4113.00
	45 yrs -	52990.22	14.70	12.88	3604.78	4114.15
	55 yrs -	47981.13	13.32	11.64	3602.19	4122.09
	65 yrs -	44243.90	12.27	10.72	3605.86	4127.23
	Total	50226.33	13.94	12.20	3603.04	4116.91
ETV	35 yrs -	41134.76	15.92	14.28	2583.84	2880.59
	45 yrs -	39068.31	15.12	13.54	2583.88	2885.40
	55 yrs -	35328.73	13.65	12.20	2588.19	2895.80
	65 yrs -	32653.20	12.57	11.22	2597.71	2910.27
	Total	37046.25	14.32	12.81	2587.03	2891.98

### Uncertainty analysis

Table 5 showed the costs and benefits of the Monte Carlo simulation were very close to the results of exact form among the group, but standard deviation of each group illustrated by the Monte Carlo simulation there was a big difference between individual patients. Cost, life expectancy, quality-adjusted life years decreased with ages, and individual differences between patients became larger. Table 6 showed the various components of the overall average and total variation. Because the different dimension of cost, life expectancy and quality-adjusted life years, we compared the degree of variation by the coefficient of variation. Table 6 showed that variability of cost, life expectancy and quality-adjusted lifeyears was similar.

**Table 5:** Expected costs and benefits of antiviral treatment for patient subgroups calculated using Monte Carlo simulation.

Group	Cost($)				Life Expectancy(years)				QALYs			
	Mean	SD	P2.5	P97.5	Mean	SD	P2.5	P97.5	Mean	SD	P2.5	P97.5
LVD												
35 yrs -	55736.39	12866.19	19017.08	69237.55	15.46	3.90	5.011	18.23	13.54	3.96	3.89	17.54
45 yrs -	52705.84	13629.44	16291.19	67618.05	14.63	4.07	4.206	18.13	12.82	4.05	3.319	17.38
55 yrs -	47767.18	15114.25	13464.28	65838.21	13.25	4.37	3.482	17.98	11.58	4.21	2.666	17.16
65 yrs -	44255.59	17381.54	9984.77	64908.20	12.27	4.99	2.453	17.89	10.71	4.7	1.917	17.07
Total	51567.39	7358.29	34266.01	62310.59	13.9	2.16	9.326	17.58	12.16	2.10	7.82	15.92
ETV												
35 yrs -	41225.05	8804.62	15701.11	58141.59	15.97	3.63	5.011	18.23	12.58	2.97	3.85	14.557
45 yrs -	39172.61	9542.21	13970.98	55608.11	15.15	3.88	4.535	18.13	11.93	3.14	3.488	14.47
55 yrs -	35356.37	10908.36	10430.53	53619.82	13.65	4.37	3.703	17.98	10.76	3.50	2.762	14.35
65 yrs -	32710.66	12611.24	8400.55	52321.01	12.58	5.03	2.453	17.89	9.92	4.0	1.885	14.27
Total	37104.14	5321.90	26056.28	46580.61	14.34	2.137	9.745	17.86	11.3	1.716	7.647	14.16

**Table 6:** Comparison of variation of cost, life expectancy and quality-adjusted lifeyears.

	Cost($)		Life Expectancy(years)		QALYs	
	Exact	Monte Carlo	Exact	Monte Carlo	Exact	Monte Carlo
**LVD**						
Mean	50226.33	51567.39	13.94	13.9	12.2	12.16
SD		7358.29		2.16		2.1
SD/Mean		0.14		0.15		0.17
**ETV**						
Mean	37046.25	37104.14	14.32	14.34	12.81	11.3
SD		5321.9		2.14		1.72
SD/Mean		0.14		0.15		0.15

LVD = lamivudine, ETV = entecavir, SD = standard deviations.

## Discussion

Clinical studies showed that antiviral therapy can inhibit the replication of hepatitis B virus, the patient virological, biochemical liver function and liver histological improvement and delay progression of liver disease. Some previous studies compared the economic effects of different antiviral therapy. ButiM[32] examined the cost-effectiveness of LVD, adefovir, telbivudine, ETV and tenofovir in patients with CHB. They concluded that tenofovir is a cost-effective strategy compared with other options for CHB. Jinghe[33] using Markov modeling conducted a cost-effectiveness analysis of LVD, telbivudine, ETV and tenofovir for CHB in Canada and concluded that compared with other therapies tenofovir generated the best results. Bin Wu [34] evaluated the economic consequences of LVD, ETV, adefovir, and telbivudine for CHB treatment in China, and concluded that ETV is the most cost-effective option when treating both HBeAg-positive and HBeAg-negative CHB patients. Astrid Wiens [35] investigated the cost-effectiveness of telbivudine and LVD for the viewpoint of the Brazilian public system, and concluded LVD is a more cost-effective or even cost-saving strategy in CHB. In our study cost-effectiveness analysis of first-line antiviral drug in Jiangsu China showed that compared with LVD, ETV was more cost-effective, consistent with study of Kenneth KC Lee [3]. We think due to the different cost of antiviral drugs and patient’s economic level it is hard to compare the result of different studies. Our study also found that the lower age the more cost-effective, suggesting that patients with CHB should accept standard treatment as soon as possible.

Development of health economic evaluation model, from the initial static decision tree model, the long-term dynamics of the Markov model, to recent years more mature synthesis and analysis of clinical evidence (such as Monte Carlo simulation and Monte Carlo model), are permeated with the application of Bayesian statistical methods. The main purpose of the application of Bayesian is uncertainty analysis, health economics evaluation model include parameter uncertainty, and focused on the cost-effectiveness analysis. Nicola J [36] applied Bayesian approach to Markov model in cost-effectiveness analyses of taxane use in advanced breast cancer, it showed using MCMC simulation methods for the synthesis of relevant evidence input into the model and the evaluation of the model itself, cost-effectiveness analysis can be implemented from a Bayesian perspective. Chao [37] applied discrete Markov process with absorbable state to the cost-utility analysis of medical intervention measures illustrated with an example of total hip prostheses, and showed that the result agreed with the current medical knowledge and clinical practice. In the study, we considered population heterogeneity uncertainty analysis obtained costs and effectiveness distribution, as well as indicated Markov model was of good robustness, of course, in practical work attention should be paid to patient variation.

## Conclusions

In conclusion, in our analysis of economic outcomes of LVD and ETV, for treating HBeAg-positive patients, ETV is the more cost-effective option. CHB patients should accept antiviral treatment as soon as possible as the lower age group the more cost-effective. The results of Monte Carlo simulation were very close to exact form and obtained the distribution of costs and effectiveness, indicated that our Markov model is of good robustness.

## Supporting Information

S1 Code WinBUGS entire code. (DOCX)
