# Supplementary material for: Application of Bayesian Approach to Cost-Effectiveness Analysis of Antiviral Treatments in Chronic Hepatitis B
Source: PLoS One. 2016 Aug 30;11(8):e0161936. doi: 10.1371/journal.pone.0161936 (PMC5004843; doi:10.1371/journal.pone.0161936)
Supplement: S1 Code — (DOCX) [file pone.0161936.s001.docx]

Exact ETV

Model 1: Closed form estimates for each strata - give results for "exact" columns in Table 4

Just load data and compile, then obtain values for C, mean.C, sd.C, BL, mean.BL, sd.BL, BQ, mean.BQ and sd.BQ using the node tool from the Info menu.

Model

model {

for(k in 1:K) { # loop over strata

# Cost and benefit equations in closed form:

####################################

# Costs

for(t in 1:N) {

ct[k,t] <- inprod(pi[k,t,], c[])/pow((1+delta.c), (t-1))

}

C[k] <- C0+sum(ct[k,])

# Benefits - life expectancy

for(t in 1:N) {

blt[k,t] <- inprod(pi[k,t,], bl[])/pow((1+delta.b), (t-1))

}

BL[k] <- sum(blt[k,])

# Benefits - QALYs

for(t in 1:N) {

bqt[k,t] <- inprod(pi[k,t,], bq[])/pow((1+delta.b), (t-1))

}

BQ[k] <- sum(bqt[k,])

# Markov model probabilities:

#######################

# Transition matrix

for(t in 2:N) {

Lambda[k,t,1,1] <- 1 - gamma30 - gamma31 - gamma32 - gamma33 - lambda[k,t]

Lambda[k,t,1,2] <- gamma30

Lambda[k,t,1,3] <- gamma31

Lambda[k,t,1,4] <- gamma32

Lambda[k,t,1,5] <- 0

Lambda[k,t,1,6] <- gamma33

Lambda[k,t,1,7] <- 0

Lambda[k,t,1,8] <- lambda[k,t]

Lambda[k,t,2,1] <- gamma34

Lambda[k,t,2,2] <- 1 - gamma34 - gamma35 - gamma36 - lambda[k,t]

Lambda[k,t,2,3] <- 0

Lambda[k,t,2,4] <- gamma35

Lambda[k,t,2,5] <- 0

Lambda[k,t,2,6] <- gamma36

Lambda[k,t,2,7] <- 0

Lambda[k,t,2,8] <- lambda[k,t]

Lambda[k,t,3,1] <- 0

Lambda[k,t,3,2] <- gamma37

Lambda[k,t,3,3] <- 1 - gamma37 - gamma38 - gamma39 - lambda[k,t]

Lambda[k,t,3,4] <- gamma38

Lambda[k,t,3,5] <- 0

Lambda[k,t,3,6] <- gamma39

Lambda[k,t,3,7] <- 0

Lambda[k,t,3,8] <- lambda[k,t]

Lambda[k,t,4,1] <- 0

Lambda[k,t,4,2] <- 0

Lambda[k,t,4,3] <- 0

Lambda[k,t,4,4] <- 1 - gamma40 - gamma41 - lambda[k,t]

Lambda[k,t,4,5] <- gamma40

Lambda[k,t,4,6] <- gamma41

Lambda[k,t,4,7] <- 0

Lambda[k,t,4,8] <- lambda[k,t]

Lambda[k,t,5,1] <- 0

Lambda[k,t,5,2] <- 0

Lambda[k,t,5,3] <- 0

Lambda[k,t,5,4] <- 0

Lambda[k,t,5,5] <- 1 - gamma42 - gamma43 - lambda[k,t]

Lambda[k,t,5,6] <- gamma42

Lambda[k,t,5,7] <- gamma43

Lambda[k,t,5,8] <- lambda[k,t]

Lambda[k,t,6,1] <- 0

Lambda[k,t,6,2] <- 0

Lambda[k,t,6,3] <- 0

Lambda[k,t,6,4] <- 0

Lambda[k,t,6,5] <- 0

Lambda[k,t,6,6] <- 1 - gamma44 - lambda[k,t]

Lambda[k,t,6,7] <- gamma44

Lambda[k,t,6,8] <-lambda[k,t]

Lambda[k,t,7,1] <- 0

Lambda[k,t,7,2] <- 0

Lambda[k,t,7,3] <- 0

Lambda[k,t,7,4] <- 0

Lambda[k,t,7,5] <- 0

Lambda[k,t,7,6] <- 0

Lambda[k,t,7,7] <- 0

Lambda[k,t,7,8] <-1

Lambda[k,t,8,1] <- 0

Lambda[k,t,8,2] <- 0

Lambda[k,t,8,3] <- 0

Lambda[k,t,8,4] <- 0

Lambda[k,t,8,5] <- 0

Lambda[k,t,8,6] <- 0

Lambda[k,t,8,7] <- 0

Lambda[k,t,8,8] <- 1

}

# Marginal probability of being in each state at time 1

pi[k,1,1] <- 1 ; pi[k,1,2]<-0 ; pi[k,1,3] <- 0 ; pi[k,1,4]<-0; pi[k,1,5] <- 0 ; pi[k,1,6]<-0 ; pi[k,1,7]<-0; pi[k,1,8]<-0

# Marginal probability of being in each state at time t>1

for(t in 2:N) {

for(s in 1:S) {

pi[k,t,s] <- inprod(pi[k,(t-1),], Lambda[k,t,,s])

}

}

}

# Mean and sd of costs and benefits over strata

#######################################

mean.C <- inprod(p.strata[], C[])

for(k in 1:4) { dev.C[k] <- pow(C[k] - mean.C, 2) }

var.C <- inprod(p.strata[], dev.C[])

sd.C <- sqrt(var.C)

mean.BL <- inprod(p.strata[], BL[])

for(k in 1:4) { dev.BL[k] <- pow(BL[k] - mean.BL, 2) }

var.BL <- inprod(p.strata[], dev.BL[])

sd.BL <- sqrt(var.BL)

mean.BQ <- inprod(p.strata[], BQ[])

for(k in 1:4) { dev.BQ[k] <- pow(BQ[k] - mean.BQ, 2) }

var.BQ <- inprod(p.strata[], dev.BQ[])

sd.BQ <- sqrt(var.BQ)

}

Data

list(N = 42, # Number of cycles

K = 4, # Number of age-sex strata

S = 8, # Number of states in Markov model

gamma30 =0.19 , # CHB to HBeAg

gamma31=0.03, # CHB to DR

gamma32=0.007, # CHB to LC

gamma33= 0.008, # CHB to HCC

gamma34= 0.03, # HBe to CHB

gamma35=0.01, # HBe to LC

gamma36 =0.003 , # HBe to HCC

gamma37 = 0.077, # DR to HBe

gamma38 = 0.04, # DR to LC

gamma39 = 0.0053, # DR to HCC

gamma40= 0.07, # LC to DLC

gamma41 = 0.034, # LC to HCC

gamma42 = 0.034, # DLC to HCC

gamma43 = 0.144 # DLC deat rate

gamma44 =0.4, # HCC deat rate

C0=12514, #ETV

c = c(14040,14040,14040, 24389,40130, 30398, 0,0), # additional costs associated with each state

bl = c(1,1,1,1,1,1,0,0), # life-expectancy benefits associated with each state (zero for death states7 and 8)

bq = c(0.795, 0.99, 0.795, 0.695, 0.661,0.672,0, 0), # QALYs associated with each state

delta.c = 0.05, # cost discount

delta.b = 0.05, # health discount

# probablilty of HBV state adjusted by age

p.strata = c(0.25, 0.25,0.25,0.25),

lambda = structure(.Data = c(0.0012216, 0.0012216,0.0012216,0.0012216,0.0012216,0.0012216,0.0012216,0.0012216,0.0031564,0.0031564,0.0031564,0.0031564,0.0031564,0.0031564,0.0031564,0.0031564,0.0031564,0.0031564,0.0031564,0.0031564,0.0031564,0.0031564,0.0031564,0.0031564,0.0075175,0.0075175,0.0075175,0.0075175,0.0075175,0.0075175,0.0075175,0.0075175,0.0075175,0.0075175,0.0075175,0.0075175,0.0075175,0.0075175,0.0075175,0.0075175,0.0232342,0.0232342,0.0031564,0.0031564,0.0031564,0.0031564,0.0031564,0.0031564,0.0031564,0.0031564,0.0075175,0.0075175,0.0075175,0.0075175,0.0075175,0.0075175,0.0075175,0.0075175,0.0075175,0.0075175,0.0075175,0.0075175,0.0075175,0.0075175,0.0075175,0.0075175,0.0232342,0.0232342,0.0232342,0.0232342,0.0232342,0.0232342,0.0232342,0.0232342,0.0232342,0.0232342,0.0232342,0.0232342,0.0232342,0.0232342,0.0232342,0.0232342,0.0232342,0.0232342,0.0075175,0.0075175,0.0075175,0.0075175,0.0075175,0.0075175,0.0075175,0.0075175,0.0232342,0.0232342,0.0232342,0.0232342,0.0232342,0.0232342,0.0232342,0.0232342,0.0232342,0.0232342,0.0232342,0.0232342,0.0232342,0.0232342,0.0232342,0.0232342,0.0232342,0.0232342,0.0232342,0.0232342,0.0232342,0.0232342,0.0232342,0.0232342,0.0232342,0.0232342,0.0232342,0.0232342,0.0232342,0.0232342,0.0232342,0.0232342,0.0232342,0.0232342,0.0232342,0.0232342,0.0232342,0.0232342,0.0232342,0.0232342,0.0232342,0.0232342,0.0232342,0.0232342,0.0232342,0.0232342,0.0232342,0.0232342,0.0232342,0.0232342,0.0232342,0.0232342,0.0232342,0.0232342,0.0232342,0.0232342,0.0232342,0.0232342,0.0232342,0.0232342,0.0232342,0.0232342,0.0232342,0.0232342,0.0232342,0.0232342,0.0232342,0.0232342,0.0232342,0.0232342,0.0232342,0.0232342,0.0232342,0.0232342,0.0232342,0.0232342

), .Dim=c(4,42))

)

Exact LAM

Model 1: Closed form estimates for each strata - give results for "exact" columns in Table 4

Model

model {

for(k in 1:K) { # loop over strata

# Cost and benefit equations in closed form:

####################################

# Costs

for(t in 1:N) {

ct[k,t] <- inprod(pi[k,t,], c[])/pow((1+delta.c), (t-1))

}

C[k] <- C0+sum(ct[k,])

# Benefits - life expectancy

for(t in 1:N) {

blt[k,t] <- inprod(pi[k,t,], bl[])/pow((1+delta.b), (t-1))

}

BL[k] <- sum(blt[k,])

# Benefits - QALYs

for(t in 1:N) {

bqt[k,t] <- inprod(pi[k,t,], bq[])/pow((1+delta.b), (t-1))

}

BQ[k] <- sum(bqt[k,])

# Markov model probabilities:

#######################

# Transition matrix

for(t in 2:N) {

Lambda[k,t,1,1] <- 1 - gamma30 - gamma31 - gamma32 - gamma33 - lambda[k,t]

Lambda[k,t,1,2] <- gamma30

Lambda[k,t,1,3] <- gamma31

Lambda[k,t,1,4] <- gamma32

Lambda[k,t,1,5] <- 0

Lambda[k,t,1,6] <- gamma33

Lambda[k,t,1,7] <- 0

Lambda[k,t,1,8] <- lambda[k,t]

Lambda[k,t,2,1] <- gamma34

Lambda[k,t,2,2] <- 1 - gamma34 - gamma35 - gamma36 - lambda[k,t]

Lambda[k,t,2,3] <- 0

Lambda[k,t,2,4] <- gamma35

Lambda[k,t,2,5] <- 0

Lambda[k,t,2,6] <- gamma36

Lambda[k,t,2,7] <- 0

Lambda[k,t,2,8] <- lambda[k,t]

Lambda[k,t,3,1] <- 0

Lambda[k,t,3,2] <- gamma37

Lambda[k,t,3,3] <- 1 - gamma37 - gamma38 - gamma39 - lambda[k,t]

Lambda[k,t,3,4] <- gamma38

Lambda[k,t,3,5] <- 0

Lambda[k,t,3,6] <- gamma39

Lambda[k,t,3,7] <- 0

Lambda[k,t,3,8] <- lambda[k,t]

Lambda[k,t,4,1] <- 0

Lambda[k,t,4,2] <- 0

Lambda[k,t,4,3] <- 0

Lambda[k,t,4,4] <- 1 - gamma40 - gamma41 - lambda[k,t]

Lambda[k,t,4,5] <- gamma40

Lambda[k,t,4,6] <- gamma41

Lambda[k,t,4,7] <- 0

Lambda[k,t,4,8] <- lambda[k,t]

Lambda[k,t,5,1] <- 0

Lambda[k,t,5,2] <- 0

Lambda[k,t,5,3] <- 0

Lambda[k,t,5,4] <- 0

Lambda[k,t,5,5] <- 1 - gamma42 - gamma43 - lambda[k,t]

Lambda[k,t,5,6] <- gamma42

Lambda[k,t,5,7] <- gamma43

Lambda[k,t,5,8] <- lambda[k,t]

Lambda[k,t,6,1] <- 0

Lambda[k,t,6,2] <- 0

Lambda[k,t,6,3] <- 0

Lambda[k,t,6,4] <- 0

Lambda[k,t,6,5] <- 0

Lambda[k,t,6,6] <- 1 - gamma44 - lambda[k,t]

Lambda[k,t,6,7] <- gamma44

Lambda[k,t,6,8] <-lambda[k,t]

Lambda[k,t,7,1] <- 0

Lambda[k,t,7,2] <- 0

Lambda[k,t,7,3] <- 0

Lambda[k,t,7,4] <- 0

Lambda[k,t,7,5] <- 0

Lambda[k,t,7,6] <- 0

Lambda[k,t,7,7] <- 0

Lambda[k,t,7,8] <-1

Lambda[k,t,8,1] <- 0

Lambda[k,t,8,2] <- 0

Lambda[k,t,8,3] <- 0

Lambda[k,t,8,4] <- 0

Lambda[k,t,8,5] <- 0

Lambda[k,t,8,6] <- 0

Lambda[k,t,8,7] <- 0

Lambda[k,t,8,8] <- 1

}

# Marginal probability of being in each state at time 1

pi[k,1,1] <- 1 ; pi[k,1,2]<-0 ; pi[k,1,3] <- 0 ; pi[k,1,4]<-0; pi[k,1,5] <- 0 ; pi[k,1,6]<-0 ; pi[k,1,7]<-0; pi[k,1,8]<-0

# Marginal probability of being in each state at time t>1

for(t in 2:N) {

for(s in 1:S) {

pi[k,t,s] <- inprod(pi[k,(t-1),], Lambda[k,t,,s])

}

}

}

# Mean and sd of costs and benefits over strata

#######################################

mean.C <- inprod(p.strata[], C[])

for(k in 1:4) { dev.C[k] <- pow(C[k] - mean.C, 2) }

var.C <- inprod(p.strata[], dev.C[])

sd.C <- sqrt(var.C)

mean.BL <- inprod(p.strata[], BL[])

for(k in 1:4) { dev.BL[k] <- pow(BL[k] - mean.BL, 2) }

var.BL <- inprod(p.strata[], dev.BL[])

sd.BL <- sqrt(var.BL)

mean.BQ <- inprod(p.strata[], BQ[])

for(k in 1:4) { dev.BQ[k] <- pow(BQ[k] - mean.BQ, 2) }

var.BQ <- inprod(p.strata[], dev.BQ[])

sd.BQ <- sqrt(var.BQ)

}

Data

list(N = 42, # Number of cycles

K = 4, # Number of age-sex strata

S = 8, # Number of states in Markov model

gamma30 =0.18 , # CHB to HBeAg

gamma31=0.1188, # CHB to DR

gamma32=0.02, # CHB to LC

gamma33= 0.008, # CHB to HCC

gamma34= 0.03, # HBe to CHB

gamma35=0.01, # HBe to LC

gamma36 =0.003 , # HBe to HCC

gamma37 = 0.077, # DR to HBe

gamma38 = 0.04, # DR to LC

gamma39 = 0.0053, # DR to HCC

gamma40= 0.07, # LC to DLC

gamma41 = 0.034, # LC to HCC

gamma42 = 0.034, # DLC to HCC

gamma43 = 0.144 # DLC deat rate

gamma44 =0.4, # HCC deat rate

C0=5397, #LAM

c = c(21157,21157,21157, 24389,40130, 30398, 0,0), # additional costs associated with each state

bl = c(1,1,1,1,1,1,0,0), # life-expectancy benefits associated with each state (zero for death states7 and 8)

bq = c(0.795, 0.99, 0.795, 0.695, 0.661,0.672,0, 0), # QALYs associated with each state

delta.c = 0.05, # cost discount

delta.b = 0.05, # health discount

# probablilty of HBV state adjusted by age

p.strata = c(0.25, 0.25,0.25,0.25),

lambda = structure(.Data = c(0.0012216, 0.0012216,0.0012216,0.0012216,0.0012216,0.0012216,0.0012216,0.0012216,0.0031564,0.0031564,0.0031564,0.0031564,0.0031564,0.0031564,0.0031564,0.0031564,0.0031564,0.0031564,0.0031564,0.0031564,0.0031564,0.0031564,0.0031564,0.0031564,0.0075175,0.0075175,0.0075175,0.0075175,0.0075175,0.0075175,0.0075175,0.0075175,0.0075175,0.0075175,0.0075175,0.0075175,0.0075175,0.0075175,0.0075175,0.0075175,0.0232342,0.0232342,0.0031564,0.0031564,0.0031564,0.0031564,0.0031564,0.0031564,0.0031564,0.0031564,0.0075175,0.0075175,0.0075175,0.0075175,0.0075175,0.0075175,0.0075175,0.0075175,0.0075175,0.0075175,0.0075175,0.0075175,0.0075175,0.0075175,0.0075175,0.0075175,0.0232342,0.0232342,0.0232342,0.0232342,0.0232342,0.0232342,0.0232342,0.0232342,0.0232342,0.0232342,0.0232342,0.0232342,0.0232342,0.0232342,0.0232342,0.0232342,0.0232342,0.0232342,0.0075175,0.0075175,0.0075175,0.0075175,0.0075175,0.0075175,0.0075175,0.0075175,0.0232342,0.0232342,0.0232342,0.0232342,0.0232342,0.0232342,0.0232342,0.0232342,0.0232342,0.0232342,0.0232342,0.0232342,0.0232342,0.0232342,0.0232342,0.0232342,0.0232342,0.0232342,0.0232342,0.0232342,0.0232342,0.0232342,0.0232342,0.0232342,0.0232342,0.0232342,0.0232342,0.0232342,0.0232342,0.0232342,0.0232342,0.0232342,0.0232342,0.0232342,0.0232342,0.0232342,0.0232342,0.0232342,0.0232342,0.0232342,0.0232342,0.0232342,0.0232342,0.0232342,0.0232342,0.0232342,0.0232342,0.0232342,0.0232342,0.0232342,0.0232342,0.0232342,0.0232342,0.0232342,0.0232342,0.0232342,0.0232342,0.0232342,0.0232342,0.0232342,0.0232342,0.0232342,0.0232342,0.0232342,0.0232342,0.0232342,0.0232342,0.0232342,0.0232342,0.0232342,0.0232342,0.0232342,0.0232342,0.0232342,0.0232342,0.0232342), .Dim=c(4,42))

)

MCMC ETV

Model 2: MC estimates for each strata - in Table 5

10000 updates

Model

model {

for(k in 1:K) { # loop over strata

# Cost and benefit equations in closed form:

####################################

# Costs

for(t in 1:N) {

ct[k,t] <- inprod(pi[k,t,], c[])/pow((1+delta.c), (t-1))

}

C[k] <- C0+sum(ct[k,])

# Benefits - life expectancy

for(t in 1:N) {

blt[k,t] <- inprod(pi[k,t,], bl[])/pow((1+delta.b), (t-1))

}

BL[k] <- sum(blt[k,])

# Benefits - QALYs

for(t in 1:N) {

bqt[k,t] <- inprod(pi[k,t,], bq[])/pow((1+delta.b), (t-1))

}

BQ[k] <- sum(bqt[k,])

# Markov model probabilities:

#######################

# Transition matrix

for(t in 1:N) {

Lambda[k,t,1,1] <- 1 - gamma30 - gamma31 - gamma32 - gamma33 - lambda[k,t]

Lambda[k,t,1,2] <- gamma30

Lambda[k,t,1,3] <- gamma31

Lambda[k,t,1,4] <- gamma32

Lambda[k,t,1,5] <- 0

Lambda[k,t,1,6] <- gamma33

Lambda[k,t,1,7] <- 0

Lambda[k,t,1,8] <- lambda[k,t]

Lambda[k,t,2,1] <- gamma34

Lambda[k,t,2,2] <- 1 - gamma34 - gamma35 - gamma36 - lambda[k,t]

Lambda[k,t,2,3] <- 0

Lambda[k,t,2,4] <- gamma35

Lambda[k,t,2,5] <- 0

Lambda[k,t,2,6] <- gamma36

Lambda[k,t,2,7] <- 0

Lambda[k,t,2,8] <- lambda[k,t]

Lambda[k,t,3,1] <- 0

Lambda[k,t,3,2] <- gamma37

Lambda[k,t,3,3] <- 1 - gamma37 - gamma38 - gamma39 - lambda[k,t]

Lambda[k,t,3,4] <- gamma38

Lambda[k,t,3,5] <- 0

Lambda[k,t,3,6] <- gamma39

Lambda[k,t,3,7] <- 0

Lambda[k,t,3,8] <- lambda[k,t]

Lambda[k,t,4,1] <- 0

Lambda[k,t,4,2] <- 0

Lambda[k,t,4,3] <- 0

Lambda[k,t,4,4] <- 1 - gamma40 - gamma41 - lambda[k,t]

Lambda[k,t,4,5] <- gamma40

Lambda[k,t,4,6] <- gamma41

Lambda[k,t,4,7] <- 0

Lambda[k,t,4,8] <- lambda[k,t]

Lambda[k,t,5,1] <- 0

Lambda[k,t,5,2] <- 0

Lambda[k,t,5,3] <- 0

Lambda[k,t,5,4] <- 0

Lambda[k,t,5,5] <- 1 - gamma42 - gamma43 - lambda[k,t]

Lambda[k,t,5,6] <- gamma42

Lambda[k,t,5,7] <- gamma43

Lambda[k,t,5,8] <- lambda[k,t]

Lambda[k,t,6,1] <- 0

Lambda[k,t,6,2] <- 0

Lambda[k,t,6,3] <- 0

Lambda[k,t,6,4] <- 0

Lambda[k,t,6,5] <- 0

Lambda[k,t,6,6] <- 1 - gamma44 - lambda[k,t]

Lambda[k,t,6,7] <- gamma44

Lambda[k,t,6,8] <-lambda[k,t]

Lambda[k,t,7,1] <- 0

Lambda[k,t,7,2] <- 0

Lambda[k,t,7,3] <- 0

Lambda[k,t,7,4] <- 0

Lambda[k,t,7,5] <- 0

Lambda[k,t,7,6] <- 0

Lambda[k,t,7,7] <- 0

Lambda[k,t,7,8] <-1

Lambda[k,t,8,1] <- 0

Lambda[k,t,8,2] <- 0

Lambda[k,t,8,3] <- 0

Lambda[k,t,8,4] <- 0

Lambda[k,t,8,5] <- 0

Lambda[k,t,8,6] <- 0

Lambda[k,t,8,7] <- 0

Lambda[k,t,8,8] <- 1

}

# Marginal probability of being in each state at time 1

pi[k,1,1] <- 1 ; pi[k,1,2]<-0 ; pi[k,1,3] <- 0 ; pi[k,1,4]<-0; pi[k,1,5] <- 0 ; pi[k,1,6]<-0 ; pi[k,1,7]<-0; pi[k,1,8]<-0

# state of each individual in strata k at time t =1

y[k,1,1:S] ~ dmulti(pi[k,1,], 1)

# state of each individual in strata k at time t > 1

for(t in 2:N) {

for(s in 1:S) {

pi[k,t,s] <- inprod(y[k,(t-1),], Lambda[k,t,,s]) # sampling probabilities

}

y[k,t,1:S] ~ dmulti(pi[k,t,], 1)

}

}

# Mean of costs and benefits over strata

#################################

mean.C <- inprod(p.strata[], C[])

mean.BL <- inprod(p.strata[], BL[])

mean.BQ <- inprod(p.strata[], BQ[])

}

Data

list(N = 42, # Number of cycles

K = 4, # Number of age-sex strata

S = 8, # Number of states in Markov model

gamma30 =0.19 , # CHB to HBeAg

gamma31=0.03, # CHB to DR

gamma32=0.007, # CHB to LC

gamma33= 0.008, # CHB to HCC

gamma34= 0.03, # HBe to CHB

gamma35=0.01, # HBe to LC

gamma36 =0.003 , # HBe to HCC

gamma37 = 0.077, # DR to HBe

gamma38 = 0.04, # DR to LC

gamma39 = 0.0053, # DR to HCC

gamma40= 0.07, # LC to DLC

gamma41 = 0.034, # LC to HCC

gamma42 = 0.034, # DLC to HCC

gamma43 = 0.144 # DLC deat rate

gamma44 =0.4, # HCC deat rate

C0=12514, #ETV

c = c(14040,14040,14040, 24389,40130, 30398, 0,0), # additional costs associated with each state

bl = c(1,1,1,1,1,1,0,0), # life-expectancy benefits associated with each state (zero for death states7 and 8)

bq = c(0.795, 0.8, 0.795, 0.695, 0.661,0.672,0, 0), # QALYs associated with each state

delta.c = 0.05, # cost discount

delta.b = 0.05, # health discount

# probablilty of HBV state adjusted by age

p.strata = c(0.25, 0.25,0.25,0.25),

lambda = structure(.Data = c(0.0012216, 0.0012216,0.0012216,0.0012216,0.0012216,0.0012216,0.0012216,0.0012216,0.0031564,0.0031564,0.0031564,0.0031564,0.0031564,0.0031564,0.0031564,0.0031564,0.0031564,0.0031564,0.0031564,0.0031564,0.0031564,0.0031564,0.0031564,0.0031564,0.0075175,0.0075175,0.0075175,0.0075175,0.0075175,0.0075175,0.0075175,0.0075175,0.0075175,0.0075175,0.0075175,0.0075175,0.0075175,0.0075175,0.0075175,0.0075175,0.0232342,0.0232342,0.0031564,0.0031564,0.0031564,0.0031564,0.0031564,0.0031564,0.0031564,0.0031564,0.0075175,0.0075175,0.0075175,0.0075175,0.0075175,0.0075175,0.0075175,0.0075175,0.0075175,0.0075175,0.0075175,0.0075175,0.0075175,0.0075175,0.0075175,0.0075175,0.0232342,0.0232342,0.0232342,0.0232342,0.0232342,0.0232342,0.0232342,0.0232342,0.0232342,0.0232342,0.0232342,0.0232342,0.0232342,0.0232342,0.0232342,0.0232342,0.0232342,0.0232342,0.0075175,0.0075175,0.0075175,0.0075175,0.0075175,0.0075175,0.0075175,0.0075175,0.0232342,0.0232342,0.0232342,0.0232342,0.0232342,0.0232342,0.0232342,0.0232342,0.0232342,0.0232342,0.0232342,0.0232342,0.0232342,0.0232342,0.0232342,0.0232342,0.0232342,0.0232342,0.0232342,0.0232342,0.0232342,0.0232342,0.0232342,0.0232342,0.0232342,0.0232342,0.0232342,0.0232342,0.0232342,0.0232342,0.0232342,0.0232342,0.0232342,0.0232342,0.0232342,0.0232342,0.0232342,0.0232342,0.0232342,0.0232342,0.0232342,0.0232342,0.0232342,0.0232342,0.0232342,0.0232342,0.0232342,0.0232342,0.0232342,0.0232342,0.0232342,0.0232342,0.0232342,0.0232342,0.0232342,0.0232342,0.0232342,0.0232342,0.0232342,0.0232342,0.0232342,0.0232342,0.0232342,0.0232342,0.0232342,0.0232342,0.0232342,0.0232342,0.0232342,0.0232342,0.0232342,0.0232342,0.0232342,0.0232342,0.0232342,0.0232342 ), .Dim=c(4,42))

)

MCMC LAM

Model 2: MC estimates for each strata in Table 5

10000 updates

Model

model {

for(k in 1:K) { # loop over strata

# Cost and benefit equations in closed form:

####################################

# Costs

for(t in 1:N) {

ct[k,t] <- inprod(pi[k,t,], c[])/pow((1+delta.c), (t-1))

}

C[k] <- C0+sum(ct[k,])

# Benefits - life expectancy

for(t in 1:N) {

blt[k,t] <- inprod(pi[k,t,], bl[])/pow((1+delta.b), (t-1))

}

BL[k] <- sum(blt[k,])

# Benefits - QALYs

for(t in 1:N) {

bqt[k,t] <- inprod(pi[k,t,], bq[])/pow((1+delta.b), (t-1))

}

BQ[k] <- sum(bqt[k,])

# Markov model probabilities:

#######################

# Transition matrix

for(t in 1:N) {

Lambda[k,t,1,1] <- 1 - gamma30 - gamma31 - gamma32 - gamma33 - lambda[k,t]

Lambda[k,t,1,2] <- gamma30

Lambda[k,t,1,3] <- gamma31

Lambda[k,t,1,4] <- gamma32

Lambda[k,t,1,5] <- 0

Lambda[k,t,1,6] <- gamma33

Lambda[k,t,1,7] <- 0

Lambda[k,t,1,8] <- lambda[k,t]

Lambda[k,t,2,1] <- gamma34

Lambda[k,t,2,2] <- 1 - gamma34 - gamma35 - gamma36 - lambda[k,t]

Lambda[k,t,2,3] <- 0

Lambda[k,t,2,4] <- gamma35

Lambda[k,t,2,5] <- 0

Lambda[k,t,2,6] <- gamma36

Lambda[k,t,2,7] <- 0

Lambda[k,t,2,8] <- lambda[k,t]

Lambda[k,t,3,1] <- 0

Lambda[k,t,3,2] <- gamma37

Lambda[k,t,3,3] <- 1 - gamma37 - gamma38 - gamma39 - lambda[k,t]

Lambda[k,t,3,4] <- gamma38

Lambda[k,t,3,5] <- 0

Lambda[k,t,3,6] <- gamma39

Lambda[k,t,3,7] <- 0

Lambda[k,t,3,8] <- lambda[k,t]

Lambda[k,t,4,1] <- 0

Lambda[k,t,4,2] <- 0

Lambda[k,t,4,3] <- 0

Lambda[k,t,4,4] <- 1 - gamma40 - gamma41 - lambda[k,t]

Lambda[k,t,4,5] <- gamma40

Lambda[k,t,4,6] <- gamma41

Lambda[k,t,4,7] <- 0

Lambda[k,t,4,8] <- lambda[k,t]

Lambda[k,t,5,1] <- 0

Lambda[k,t,5,2] <- 0

Lambda[k,t,5,3] <- 0

Lambda[k,t,5,4] <- 0

Lambda[k,t,5,5] <- 1 - gamma42 - gamma43 - lambda[k,t]

Lambda[k,t,5,6] <- gamma42

Lambda[k,t,5,7] <- gamma43

Lambda[k,t,5,8] <- lambda[k,t]

Lambda[k,t,6,1] <- 0

Lambda[k,t,6,2] <- 0

Lambda[k,t,6,3] <- 0

Lambda[k,t,6,4] <- 0

Lambda[k,t,6,5] <- 0

Lambda[k,t,6,6] <- 1 - gamma44 - lambda[k,t]

Lambda[k,t,6,7] <- gamma44

Lambda[k,t,6,8] <-lambda[k,t]

Lambda[k,t,7,1] <- 0

Lambda[k,t,7,2] <- 0

Lambda[k,t,7,3] <- 0

Lambda[k,t,7,4] <- 0

Lambda[k,t,7,5] <- 0

Lambda[k,t,7,6] <- 0

Lambda[k,t,7,7] <- 0

Lambda[k,t,7,8] <-1

Lambda[k,t,8,1] <- 0

Lambda[k,t,8,2] <- 0

Lambda[k,t,8,3] <- 0

Lambda[k,t,8,4] <- 0

Lambda[k,t,8,5] <- 0

Lambda[k,t,8,6] <- 0

Lambda[k,t,8,7] <- 0

Lambda[k,t,8,8] <- 1

}

# Marginal probability of being in each state at time 1

pi[k,1,1] <- 1 ; pi[k,1,2]<-0 ; pi[k,1,3] <- 0 ; pi[k,1,4]<-0; pi[k,1,5] <- 0 ; pi[k,1,6]<-0 ; pi[k,1,7]<-0; pi[k,1,8]<-0

# state of each individual in strata k at time t =1

y[k,1,1:S] ~ dmulti(pi[k,1,], 1)

# state of each individual in strata k at time t > 1

for(t in 2:N) {

for(s in 1:S) {

pi[k,t,s] <- inprod(y[k,(t-1),], Lambda[k,t,,s]) # sampling probabilities

}

y[k,t,1:S] ~ dmulti(pi[k,t,], 1)

}

}

# Mean of costs and benefits over strata

#################################

mean.C <- inprod(p.strata[], C[])

mean.BL <- inprod(p.strata[], BL[])

mean.BQ <- inprod(p.strata[], BQ[])

}

Data

list(N = 42, # Number of cycles

K = 4, # Number of age-sex strata

S = 8, # Number of states in Markov model

gamma30 =0.18 , # CHB to HBeAg

gamma31=0.1188, # CHB to DR

gamma32=0.02, # CHB to LC

gamma33= 0.008, # CHB to HCC

gamma34= 0.03, # HBe to CHB

gamma35=0.01, # HBe to LC

gamma36 =0.003 , # HBe to HCC

gamma37 = 0.077, # DR to HBe

gamma38 = 0.04, # DR to LC

gamma39 = 0.0053, # DR to HCC

gamma40= 0.07, # LC to DLC

gamma41 = 0.034, # LC to HCC

gamma42 = 0.034, # DLC to HCC

gamma43 = 0.144 # DLC deat rate

gamma44 =0.4, # HCC deat rate

C0=5397, #LAM

c = c(21157,21157,21157, 24389,40130, 30398,0,0), # additional costs associated with each state

bl = c(1,1,1,1,1,1,0,0), # life-expectancy benefits associated with each state (zero for death states7 and 8)

bq = c(0.795, 0.99, 0.795, 0.695, 0.661,0.672,0, 0), # QALYs associated with each state

delta.c = 0.05, # cost discount

delta.b = 0.05, # health discount

# probablilty of HBV state adjusted by age

p.strata = c(0.25, 0.25,0.25,0.25),

lambda = structure(.Data = c(0.0012216, 0.0012216,0.0012216,0.0012216,0.0012216,0.0012216,0.0012216,0.0012216,0.0031564,0.0031564,0.0031564,0.0031564,0.0031564,0.0031564,0.0031564,0.0031564,0.0031564,0.0031564,0.0031564,0.0031564,0.0031564,0.0031564,0.0031564,0.0031564,0.0075175,0.0075175,0.0075175,0.0075175,0.0075175,0.0075175,0.0075175,0.0075175,0.0075175,0.0075175,0.0075175,0.0075175,0.0075175,0.0075175,0.0075175,0.0075175,0.0232342,0.0232342,0.0031564,0.0031564,0.0031564,0.0031564,0.0031564,0.0031564,0.0031564,0.0031564,0.0075175,0.0075175,0.0075175,0.0075175,0.0075175,0.0075175,0.0075175,0.0075175,0.0075175,0.0075175,0.0075175,0.0075175,0.0075175,0.0075175,0.0075175,0.0075175,0.0232342,0.0232342,0.0232342,0.0232342,0.0232342,0.0232342,0.0232342,0.0232342,0.0232342,0.0232342,0.0232342,0.0232342,0.0232342,0.0232342,0.0232342,0.0232342,0.0232342,0.0232342,0.0075175,0.0075175,0.0075175,0.0075175,0.0075175,0.0075175,0.0075175,0.0075175,0.0232342,0.0232342,0.0232342,0.0232342,0.0232342,0.0232342,0.0232342,0.0232342,0.0232342,0.0232342,0.0232342,0.0232342,0.0232342,0.0232342,0.0232342,0.0232342,0.0232342,0.0232342,0.0232342,0.0232342,0.0232342,0.0232342,0.0232342,0.0232342,0.0232342,0.0232342,0.0232342,0.0232342,0.0232342,0.0232342,0.0232342,0.0232342,0.0232342,0.0232342,0.0232342,0.0232342,0.0232342,0.0232342,0.0232342,0.0232342,0.0232342,0.0232342,0.0232342,0.0232342,0.0232342,0.0232342,0.0232342,0.0232342,0.0232342,0.0232342,0.0232342,0.0232342,0.0232342,0.0232342,0.0232342,0.0232342,0.0232342,0.0232342,0.0232342,0.0232342,0.0232342,0.0232342,0.0232342,0.0232342,0.0232342,0.0232342,0.0232342,0.0232342,0.0232342,0.0232342,0.0232342,0.0232342,0.0232342,0.0232342,0.0232342,0.0232342), .Dim=c(4,42))

)
